# 

**Published:** 1841-10

**Authors:** 


### CONTENTS OF No. XXIV.

OF THE

### British and Foreign Medical Review.

OCTOBER, 1841.

---

### PART FIRST.—Analytical and Critical Reviews.

PAGE

ART. I.—1. On the Typhus Fever which occurred at Philadelphia in the spring and summer of 1836; illustrated by Clinical Observations at the Philadelphia Hospital; showing the distinction between this form of disease and Dothinenteritis or the Typhoid Fever with alteration of the follicles of the small intestine. By W. W. GERHARD, M.D. . . . . . 293

2. Considérations sur la Fièvre Typhoide, &c. &c.—Du Typhus Fever et de la Fièvre Typhoide d'Angleterre. Par M. VALLEIX, M.D. . .. ib.

On the Typhoid and Typhus Fever of France and England. By M. VALLEIX, M.D.

3. Report on the Epidemic Fever of Edinburgh. An Account of the Symptoms and Treatment. By WILLIAM HENDERSON, F.R.C.PH. &c. . . ib.

Analysis and Details of 47 Inspections after Death. By JOHN REID, F.R.C.PH. &c.

4. Recherches Anatomiques, Pathologiques et Thérapeutiques sur la Maladie connue sous les noms de Fièvre typhoïde, putride, adynamique, ataxique, bilieuse, muqueuse, gastro-entérite, entérite folliculeuse, dothinentérie, &c., comparée avec les maladies aigues les plus ordinaires. Par P.C.A. LOUIS, M.D. ib.

Anatomical, Pathological, and Therapeutical Researches on the Disease known under the names of Tpphoid Fever, &c. By P. C. A. LOUIS.

ART. II.—Guy's Hospital Reports. Nos. X-XI-XII. April and October, 1840, and April, 1841 . . . . . . . 326

ART. III.—1. Maladies des Enfans, Affections de Poitrine. Première Partie, Pneumonie. Par MM. RILLIET et BARTHEZ . . . . 350

A Treatise on the Diseases of Children. Affections of the Chest. First Part, Pneumonia. By Messrs. RILLIET and BARTHEZ.

2. Recherches sur la Bronchite capillaire, purulente et pseudo-membraneuse, (Catarrhe suffocant, Croup bronchique,) chez les Enfants. Par S.A.FAUVEL, M.D. ib.

Researches on the purulent and pseudo-membranous form of Capillary Bronchitis in Children, (otherwise called suffocative Catarrh, or bronchial Croup.) By S. A. FAUVEL, D.M.P.

3. Ueber die acute Bronchitis der Kinder, und ihr Verhältniss zu den verwandten Krankheitsformen. Von Dr. W. CRUSE, &c. . . . . ib.

On the acute Bronchitis of Children, and its Relation to similar Diseases. By Dr. W. CRUSE, &c.

4. Ein Beitrag zur Lehre von der Bronchitis der Kinder. Von Dr. R. KUTTNER ib.

An Essay on the Bronchitis of Children. By Dr. KUTTNER.

ART. IV.—1. Précis théorique et pratique sur les Maladies vénériennes. Par P. BAUMÈS . . . . . . . . 362

A Theoretical and Practical Treatise on Venereal Diseases. By P. BAUMÈS.

2. A complete Practical Treatise on Venereal Diseases, and their immediate and remote consequences; including observations on certain affections of the uterus, attended with discharges. By WILLIAM ACTON . . ib.

ART. V.—Ontleedkundig Onderzoek, Beschrijving en Rangschikking der Dubbelde Missgeboorten. Door W. VROLIK, M.D. . . . . 374

Anatomical Examination, Description, and Arrangement of Double Monsters. By W. VROLIK, M.D.

ART. VI.—Nouvelles Considérations sur les Rétentions d'Urine, suivies d'un Traité sur les Calculs urinaires, sur la manière d'en connaitre la nature dans l'intérieure de la Vessie, et la possibilité d'en opérer la destruction sans l'opération de la Taille. Par le Dr. CIVIALE . . . . . 388

13. A Treatise on Stricture of the Urethra, with an Appendix on Dilatation by Fluid Pressure, &c. By JAMES ARNOTT, M.D. . . . . . ib.

ART. VII.—A Practical Essay on some of the principal Surgical Diseases of India. By F. H. BRETT . . . . . . 422

§

PAGE

ART. VIII.—Mittheilungen aus dem Archiv der Gesellschaft pratischer Aerzte zu Riga. Erste Sammlung . . . . . . 430
Communications from the Archives of the Society of Physicians practising in Riga. First Collection.

ART. IX.—An Account of the Yellow Fever which appeared in the City of Galveston, Republic of Texas, in the Autumn of 1839, with Cases and Dissections. By ASHBEL SMITH, M.D. A.M. . . . . . 433

ART. X.—Observations on the Surgical Pathology and Treatment of Aneurism. By WILLIAM HENRY PORTER, A.M. . . . . . 438

ART. XI.—Leçons Orales de Clinique Chirurgicale faites à l'Hôpital de la Charité, par M. le Professeur Velpeau. Par le Docteur P. PAVILLON . 452
Clinical Lectures on Surgery delivered at the Hospital of La Charité, by Professor Velpeau. By Dr. P. PAVILLON.

ART. XII.—1. Library of Medicine: Vol. VI.—A System of Midwifery. By Dr. RIGBY 461
2. The Principles and Practice of Obstetric Medicine and Surgery, in reference to the Process of Parturition; with One Hundred Illustrations on Steel and Wood. By FRANCIS H. RAMSBOTHAM, M.D. . . . . . ib.
3. The Elements of Obstetric Medicine, with the Description and Treatment of some of the principal Diseases of Children. By DAV. D. DAVIS, M.D. M.R.S.L. ib.

ART. XIII.—Three Memoirs on the Development and Structure of the Teeth and Epithelium, read at the Ninth Annual Meeting of the British Association in August 1839; with the Diagrams exhibited in illustration of them. By ALEXANDER NASMYTH, F.L.S. F.G.S. &C. . . . . 491

ART. XIV.—Die Venerische Krankheit der Pferde. Von J. L. HARTHAUSEN . 494

The Venereal Disease of the Horse. By Dr. J. L. HARTHAUSEN.

ART. XV.—Om der Sanitaire Forholde i Fængsler efter nyere Systemer. Ved Professor FRED. HOLST, M.D. . . . . . . 498
On the Influence of the Systems recently adopted in various Prisons upon the Health of their Inmates. By Professor FRED. HOLST, M.D.

ART. XVI.—A Treatise on Pyrosis Idiopathica, or Water-brash, as contrasted with certain forms of Indigestion and Organic Lesions of the Abdominal Organs; together with the Remedies, Dietetic and Medicinal. By T. WEST, M.D. . 503

ART. XVII.—On Gout: its Cause, Nature, and Treatment. By JOHN PARKIN, &C. 507

ART. XVIII.—An Essay on the Chemical, Botanical, Physical, and Parturient Properties of the Secale Cornutum: with an Engraving. By T. H. WARDLEWORTH 509

---

### PART SECOND.—Bibliographical Notices.

ART. I.—The Surgical Anatomy of Inguinal Herniæ, the Testis and its Coverings. By THOMAS MORTON . . . . . . 511

ART. II.—De la Compression contre les Tumeurs Blanches des parties dures. Par le Docteur De LAVACHERIE . . . . . 512
Memoir on the Treatment of White Swellings by Compression. By Professor LAVACHERIE.

ART. III.—The Principles of Physiology applied to the Preservation of Health and to the Improvement of Physical and Mental Education. By A. COMBE, M.D. 513

ART. IV.—Traités et Découvertes sur la Physiologie de la Moelle Espinière. Par J. VAN DEEN, M.D. . . . . . . 514
Essays and Discoveries on the Physiology of the Spinal Marrow. By J. VAN DEEN.

ART. V.—Observations on the Structure and Diseases of the Testis. By Sir ASTLEY COOPER, Bart., F.R.S. . . . . . 515

ART. VI.—Over de Indische Sprouw (apthæ orientales.) Door W. BOSCH . 516

On the Indian Thrush. By W. BOSCH.

ART. VII.—A New Operation for the Cure of Amaurosis, Impaired Vision, and Shortsightedness. By JAMES J. ADAMS, F.L.S. G.S. &C. . . 517

ART. VIII.—The Surgeon's Vade Mecum. By ROBERT DRUITT . . 518

ART. IX.—Researches into the Physical History of Mankind. By Dr. PRICHARD 519

ART. X.—The Philosophy of Mystery. By W. C. DENDY . . . ib.

ART. XI.—An Enquiry concerning the Diseases and Functions of the Brain, the Spinal Cord, and the Nerves. By AMARIAH BRIGHAM, M.D. . . 520

ART. XII.—The Cure of Strabismus by Surgical Operation. By W. MACKENZIE, M.D. ib.

PAGE

ART. XIII.—Du Système Nerveux de la Vie Animale, et de la Vie Végétative; de leur Connexions Anatomiques, et des Rapports Physiologiques, Psychologiques, et Zoologiques, qui existent entre eux. Par A. BAZIN, D.M.P. . 521
On the Nervous Systems of Animal and of Vegetative Life, their Anatomical Connexions, and their Physiological, Psychological, and Zoological Relations. By A. BAZIN, D.M.P.
ART. XIV.—A Series of Anatomical Sketches and Diagrams. By T. WORMALD, and A. M. M'WHINNIE . . . . . ib.
ART. XV.—A new Process for Purifying the Waters supplied to the Metropolis by the existing Water Companies. By THOMAS CLARKE . . . 522
ART. XVI.—Tic Douloureux, or Neuralgia Facialis, and other Nervous Affections; their Seat, Nature, and Cause; with Cases Illustrating Successful Methods of Treatment. By A. H. ALNATT, M.D. A.M. . . . . 524

---

### PART THIRD.—Selections from the British and Foreign Journals.

### I. THE FOREIGN JOURNALS.

#### ANATOMY AND PHYSIOLOGY.

Dr. HEINE on the Organic Causes of the Motion of the Heart . . 525
Dr. KUERSCHNER on the Stroke of the Heart . . . . 526
GRANDONI's Experiments to determine if Portions cut from Leeches are reproduced 527
Prof. BISCHOFF's Microscopic Examination of Lymph . . . 528
Dr. LETELLIER's Microscopic Experiments on Blood, Plastic Lymph, Pus, and Milk ib.
Dr. HOPPE on Spontaneous Vomiting after Division of the Nervi Vagi . . ib.
M. VALENCIENNES on Development of Heat during Incubation of Oviparous Reptiles 529
Dr. GADDI on the Ramifications of Minute Arteries and Veins in the Intestines . ib.
Dr. FOURNIER on the Presence of Cystierci in a Tumour having appearance of a Boil 530
VALENTIN and WILL on the Temperature of Marine Invertebrata . . 531
M. COSTER's Experiments on Tubercles, and Ferruginous Bread in preventing them 531
Dr. JACOBS on Spontaneous Combustion of the Human Body . . ib.
Professor GRIMELLI on Injections of the Iris . . . . 532
M. PERSOZ on the Oxidation of Gelatine . . . . ib.
Prof. CRUVEILHIER on the Motions and Sounds of the Heart . . . 534

#### PATHOLOGY, PRACTICAL MEDICINE, AND THERAPEUTICS.

M. PRUS's Cases illustrating the Pathology of the Spinal Marrow . . 535
M. BOURDON on Puerperal Fever in the Hôtel Dieu at Paris in 1840 . . 536
Dr. KESSLER on Inflammation of the Mouth, Pharynx, Œsophagus, and Stomach . 537
Dr. HOLSCHER on Ileus from Hypertrophy of the Pancreas . . . 538
M. LEBLANC, Strangulation produced by a Fatty Tumour of the Mesentery of a Mare ib.
Dr. FRECHIER on Epidemic Hemeralopia in the Department Bouches-du Rhône . 538
M. DONNÉ on the Composition of the Urine in Pregnancy and under Disease . 539
Dr. NARDO on the Use of Oxalic Acid . . . . . ib.
G. NEGRIER's Treatment of Scrofulous Affections by preparations of Walnut Leaves ib.
Dr. FORCKE's Intussusception and Separation of a part of the Ileum . . 540
Dr. WEHR's Case of Deficiency of the Uterus . . . . ib.
M. HUGUIER on Syphilis in Pregnant and recently-delivered Females . . 541

#### SURGERY.

M. BÉRARD on Absence of the Nasal Duct, and its Artificial Formation . ib.
M. LEPAGE's New Artificial Leg . . . . . ib.
Dr. LERCHE on the Influence of Galvanism in Organic Diseases of the Eye . 542
M. HELIOT on M. Ricord's New Urethro-plastic Operation . . . 543
M. TAVIGNOT on Application of Oil-varnish in Fractures of the Limbs of Children 544
Dr. HELLO on Excision of the Head of the Humerus after Compound Fracture . 545
M. DUMEAUX on a new variety of Hernia: Hernia destitute of Peritoneal Sac . 546
M. BEYDLER's Two Cases of Strabismus cured without Operation . . ib.
M. DUBOURG on the Radical Cure of Spina Bifida, by a New Operation . ib.
Dr. RUETE's Improved Method of Scleroticonyxis . . . . 548

PAGE

Dr. Oppler's Case of Pruritus Scroti cured by fresh Lemon Juice . . ib.
Dr. Troschel on the Condition of the Nails in Fractures of the Limbs . . ib.
Dr. Detmold on the Cure of Ozæna . . . . . . 549
Dr. Stilling on Hairs within the Eye . . . . . . ib.
M. Jobert on the Employment of Nitrate of Silver in White Swellings . . 550
Dr. Maslieurat Lagemard on Ecchymosis of the Eye and Eyelids . . ib.
M. V. Morel on Dislocations of the Sternal Extremity of the Clavicle backwards 551
M. Gautric's Case of Internal Intestinal Strangulation . . . ib.
Traumatic Pneumothorax, produced by violent pressure on the Chest . . 552
MM. Thillage and Berard's Report on M. Louvrier's Treatment of Anchylosis ib.
MM. Pravaz, Bonnet, Guerin, &c. on the Action of the Muscles of the Eye . 553
MM. Velpeau and Bonnet on the Operations for Stammering in France . . 554
M. J. Guerin's Application of the Subcutaneous Method for Strangulated Hernia 556
M. Velpeau on the Ligature of the Temporal and Facial Arteries in Epilepsy . ib.
Dr. Mignot on the Effects of Extract of Belladonna in reducing Paraphimosis . ib.
MM. Rousseau and Serrurier on Development of Cryptogamia of Vertebrata . 557
M. Civiale on Contraction of the Vesical Orifice of the Urethra . . ib.

### MIDWIFERY, AND DISEASES OF WOMEN AND CHILDREN.

Dr. Wehr on the Secretion of Milk after Menstruation . . . 558
Prof. Osiander on Tubercles in the Uterus as a cause of difficult Labour . . ib.
Dr. Münchmeyer on Cold Affusion in the Treatment of Acute Hydrocephalus . 559
Dr. Scholer on the Immersion of Children apparently still-born in Cold Water . 560

### MEDICAL JURISPRUDENCE AND TOXICOLOGY.

Dr. Krügelstein on Death by Hanging, with numerous Wounds on the Person . ib.
B. M. Tavignot on Poisoning by a Tobacco Enema . . . . 562
Medico-legal question on the criminal production of Abortion in a Monster . ib.

### ANIMAL CHEMISTRY AND PHARMACY.

M. Fremy on the Chemical Composition of the Brain . . . . 563
M. Liebeg on the Identical Composition of Fibrin and Albumen . . . ib.

### II. THE AMERICAN AND COLONIAL JOURNALS.

Origin of the Operation for Strabismus . . . . 564
T. T. Smiley and J. M. Wallace on Cases of Poisoning by Arsenic . . ib.
J. J. Ridley's Anomalous Case of Periodical Suspended Perception . . 565
W. M. M'Pherters and J. C. Perry on Kiesteine as a Test of Pregnancy . 566
J. C. Perry on the Use of the Chloride of Silver . . . . 567

### III. THE BRITISH JOURNALS.

J. C. August Franz's Case of a Gentleman born Blind, with Observations . ib.
M. Donovan on the Preparation of Vinum Ferri . . . . 568
M. Donovan's Hints to Prescribers of Sulphate of Quinina . . . 569
M. Barry on the Chorda Dorsalis . . . . . 570
M. Barry on the Corpuscles of the Blood . . . . ib.
J. Blake on the Action of Inorganic Compounds on the Blood . . . 571
J. Toynbee on Non-Vascularity of Animal Tissues, their Organization and Nutrition 572

### Part Fourth.—Medical Intelligence.

New Bills of Mortality for London . . . . . 575
Royal College of Surgeons in London . . . . . 576
The Metropolitan Convalescent Institution . . . . ib.
Books received for Review . . . . . . 577
Index, Title, &c.

